# Comparison of the acute effects of Tai chi versus high-intensity interval training on inhibitory control in individuals with substance use disorder

**DOI:** 10.3389/fpsyg.2022.941719

**Published:** 2022-10-04

**Authors:** Yanqiang Yin, Suyong Yang, Ke Xiao, Tianyuan Wang, Jiabin Wang, Wolfgang I. Schöllhorn, Dong Zhu, Xiaowu Pang

**Affiliations:** ^1^Department of PE and Military Training, Zhejiang Sci-Tech University, Hangzhou, China; ^2^School of Psychology, Shanghai University of Sport, Shanghai, China; ^3^Department of Physiological Rehabilitation, Shanghai Drug Administration, Shanghai, China; ^4^College of Wushu, Shanghai University of Sport, Shanghai, China; ^5^Department of Training and Movement Science, Johannes Gutenberg-University Mainz, Mainz, Germany; ^6^School of International Education, Shanghai University of Sport, Shanghai, China; ^7^Department of Physical Education, Hangzhou Medical College, Hangzhou, China

**Keywords:** Tai chi, high-intensity interval training, acute exercise, substance use disorder, inhibitory control

## Abstract

**Objective:**

The purpose of this study is to investigate the effects of a single session of Tai chi (TC) exercise and high-intensity interval training (HIIT) on inhibitory control in individuals with substance use disorder (SUD).

**Methods:**

A total 47 males with methamphetamine dependence were recruited from a compulsory drug rehabilitation center; participation in this study was voluntary. The participants were randomly assigned to the TC group or the HIIT group, and computer-based Go/No-go and Stroop tasks were used to assess inhibitory control in an indoor setting prior to and following exercise. Independent sample *t*-test was applied for baseline comparison of continuous variables, while analysis of variance was applied to test differences in the effect of each intervention before and after a single session of exercise.

**Results:**

In Go/No-go test, the reaction time of the TC and HIIT groups in the post-test was shorter than that at the baseline, and the response accuracy of the post-test were higher than that of the baseline. In the Stroop task, the reaction time of two groups in the post-test was shorter than that at the baseline; while, greater improvement in response accuracy was observed in HIIT group in the post-test than that of the baseline.

**Conclusion:**

Both TC and HIIT can promote inhibitory control in individuals with SUD. Compared with the TC group, the HIIT group showed greater improvements in response accuracy. These findings demonstrate the potential of TC and HIIT in improving cognition in SUD.

**Clinical trial registration:**

[http://www.chictr.org.cn/], identifier [ChiCTR1900022158].

## Introduction

As a global public health problem (Whiteford et al., 2015), substance use disorder (SUD) has elicited increasing concern. Complex reasons may explain why individuals with SUD are difficult to cure. Numerous studies have demonstrated that SUD or drug addiction is a chronic and relapsing brain disease in which addicted patients continually exhibit drug-seeking behavior despite its adverse consequences for their psychology and physiology (Tai and Volkow, 2013; Bellamoli et al., 2014; Borjkhani et al., 2018). Neuropsychological studies have verified that the cognitive function of individuals exposed to certain substances (e.g., drugs, alcohol, cigarettes) for extended periods of time show significant impairment that can be associated with abnormal brain function and metabolism caused by substance addiction (Scott et al., 2007; D’Souza, 2019). Many studies have shown that the executive function of SUD patients is severely impaired (Henry et al., 2011). Within the scope of cognitive function, executive function is a key psychological process composed of inhibition (Miyake et al., 2000; Lehto et al., 2011). And inhibitory control, as the core component of executive function (Miyake et al., 2000; Lehto et al., 2011), its impairment is often reported in SUD patients (Feil et al., 2011). The cognitive impairment of individuals with SUD is mainly manifested by their reduction of impulse control and associated decision-making compared with the normal population (Smith et al., 2014; Jones et al., 2016). Compared with assessments obtained after recent methamphetamine (Meth) use, the impulsivity self-reported by Meth dependent was higher following about 1 week of abstinence from drugs (Jones et al., 2016). Elevated impulsivity and impaired cognition of decision-making have been reported in persons with heavy drug use; both conditions are strongly state-dependent in the SUD population and may be suitable for monitoring treatment success (Quednow et al., 2007; Hulka et al., 2015). In addition, a number of studies have confirmed the presence of learning and memory disorders in individuals with SUD, and these disorders have been proposed to increase the vulnerability of the latter to drug addiction (Almeida et al., 2017; Wunderli et al., 2017). That is to say, learning and memory defects render individuals likely to become addicted to drugs and increase the difficulty of abstinence. Therefore, improving the cognitive function of individuals with SUD can be assumed to play a vital role in drug rehabilitation.

Physical exercise has been strongly recommended as a rehabilitation program for SUD (Lynch et al., 2013; D’Souza, 2019) and has been proven to be a positively effective means of drug rehabilitation (Swenson et al., 2020). Numerous studies have assessed the effects of short exercise sessions and long-term exercise protocols as an adjunct therapy for SUD. The majority of these studies focus on physical and mental health, withdrawal symptoms, craving, and duration of abstinence (Roberts et al., 2012; Peterson et al., 2014; Wang et al., 2014; Zhu et al., 2016, 2018; Ashdown-Franks et al., 2020). The impacts of exercise on cognitive psychology and brain neuroscience have received growing interest in efforts to explore the mechanism of drug addiction. Studies have shown that exercise can improve age-related declines in cerebral blood flow, which is thought to be related with the improvement with brain health and executive function (Lucas et al., 2012). The beneficial effects of long-term exercise on cognition have been demonstrated (Gimenez-Meseguer et al., 2020; Swenson et al., 2020), and even a single session of exercise (i.e., acute exercise) has been consistently shown to exert a positive influence on cognitive function (Chang et al., 2012). Acute exercise effectively improves inhibitory control in individuals with SUD (Wang et al., 2015). However, despite the numerous positive effects of exercise on the human body, systematic research on the application of this therapy to individuals with SUD remains at an early stage. Studies in this area fall below the strictest criteria of scientific rigor, and the related mechanisms and modalities have yet to be thoroughly investigated (Colledge et al., 2018). Most available studies solely explore the effectiveness of aerobic or moderate-intensity exercise on the physical attributes, mental recovery, and brain function of patients with SUD. While previous studies have shown benefits in motoric and cognitive domains, how these effects are functionally related remains unclear. The effectiveness of different doses of specific exercise parameters (e.g., intensity) must also be investigated (Roberts et al., 2012), as this topic is seldom discussed in research on exercise rehabilitation for individuals with SUD (Wang et al., 2016). Some studies have explored the effects of moderate- and high-intensity exercise, as well as aerobic and anaerobic forms of exercise, on drug rehabilitation (Flemmen et al., 2014; Colledge et al., 2018). However, the single-feature (e.g., moderate intensity) exercise protocols used therein (Wang et al., 2016) do not allow precise exercise prescriptions to be developed for individuals with SUD.

Tai chi (TC), a traditional Chinese sport, has been increasingly applied to the field of drug rehabilitation. Previous studies reported that TC can be beneficial to the physical and mental health of individuals with SUD (Zhu et al., 2016, 2018), but how TC affects the cognition of SUD is not completely understood. A large number of studies have demonstrated that TC can improve cognition in the elderly and cognitive-impairment groups (Northey et al., 2018). The cognitive impairment of individuals with SUD has been confirmed (Scott et al., 2007), and the results provide indications for the improvement of cognitive function, especially the improvement of inhibitory control, are related to drug abstinence (Almeida et al., 2017; Wunderli et al., 2017). A greater understanding of the cognitive impact of TC on patients with SUD is warranted.

Depending on its style, TC is described as a form of physical exercise with low to moderate intensity (Pan et al., 2013; Hawkes et al., 2014; Zhu et al., 2016). In general, a TC style with moderate intensity is believed to assist detoxification, relieve withdrawal syndrome, and inhibit relapse impulses and behaviors; it is also considered relatively safe (McGregor et al., 2016; Onerup et al., 2017; Zhao et al., 2018). Moderate-intensity continuous training (MICT) is widely used in drug rehabilitation programs (Gimenez-Meseguer et al., 2020). The effects of high-intensity interval training (HIIT) on individuals with SUD have received great attention (Flemmen et al., 2014) as HIIT is considered a time-saving, highly efficient, and economical form of exercise (Gibala et al., 2012). HIIT is characterized by a short series of high-intensity repetitions interspersed with low-intensity recovery phases. However, increased exercise intensity is widely believed to result in greater risk of adverse events, such as muscle injury and systemic inflammation (Thompson et al., 2007; Schnyder and Handschin, 2015; Yang and Hu, 2018). The application of high-intensity exercise to drug dependents is considered a critical endeavor because patients with SUD are not normally in excellent physical condition (Zhu et al., 2016, 2018) and participation in high-intensity sport features an inherent risk of sports-related injuries (Read et al., 2015). Nevertheless, several long- and short-term studies argue that HIIT has the same value as, if not is better than, MICT in terms of improving body composition, cardiovascular function, metabolic health tolerance, safety, and exercise enjoyment and adherence (Helgerud et al., 2007; Macpherson et al., 2011; Currie et al., 2013; Gayda et al., 2016; Gillen et al., 2016; Heisz et al., 2016). More importantly, some authors have emphasized that HIIT is beneficial in reducing cardiovascular risk and indirectly improving mortality risk (Kodama et al., 2009; Flemmen et al., 2014). Unlike TC or other moderate exercise, HIIT may require more exercise monitoring and specific patterns to reduce potential risks because high-intensity exercise may be challenging for the SUD population. Different exercise intensities or forms may lead to different cognitive benefits. Sport-physiological research supports the assumption that compared with moderate-intensity exercise, higher-intensity exercise could lead to greater cognitive benefits (Oberste et al., 2017). Many studies have indicated HIIT has a positive effect on improving cognition in the elderly and cognitive-impairment groups (Lautenschlager et al., 2008; Baker et al., 2010; Fiorelli et al., 2019). Currently, the evidence in favor of high-intensity training for SUD is weak. Few studies have discussed the cognitive rehabilitation of patients with SUD through high-intensity training (Flemmen et al., 2014; Wang et al., 2016; Chen et al., 2020). Consequently, the impact of HIIT on the cognition of patients with SUD is unclear and requires further research.

Whether different forms or intensities of exercise exert different effects on substance use-related outcomes must be determined. The theoretical basis of TC and HIIT beside the cognitive treatment of SUD has not been fully confirmed in the scientific literature. Given the benefits of TC and HIIT in physical and cognitive rehabilitation in different groups, this study seeks to explore which form of exercise is more suitable for the cognitive rehabilitation of SUD. The purpose of this study is to compare the effects of TC and HIIT on the recovery of cognitive inhibitory control in patients with SUD by using specific cognitive tests that are typically associated with executive functions. And the study hypothesized that HIIT may have better benefits for inhibitory control.

## Materials and methods

### Design

This single (assessor)-blind, two-group randomized controlled trial was conducted to compare the acute effects of TC and HIIT practice. The study was carried out from November 2019 to December 2019 and included a single session of TC or HIIT intervention. The primary outcome was measured at the baseline and after each session. All exercise interventions and tests were conducted in a laboratory environment. Participants were recruited from a 1-year follow-up trial including individuals participating in either high-intensity exercise (i.e., running, resistance training, rope skipping) or TC (Zhu et al., 2016) for at least 6 months in a Shanghai compulsory rehabilitation center (SCRC). The frequency of exercise was three times a week, and each session was conducted for 1 h. The study protocol was approved by the Shanghai Narcotics Control Commission Ethical Committees of and the Shanghai University of Sport. All participants signed written informed consent forms prior to participating in this study.

### Participants

The participants were male Meth-dependent individuals. 47 eligible Meth-dependent individuals recruited from 120 individuals with SUD were receiving treatment in an SCRC voluntarily participated in this study. Participants were in abstinence throughout treatment in the SCRC. The inclusion criteria were: (1) age 18–40 years, (2) met the diagnosis of Meth dependence according to Diagnostic and Statistical Manual of Mental Disorders criteria (DSM-IV), (3) treatment duration in the SCRC exceeding 1 year, (4) no serious mental illness, and (5) completed primary school or a higher level of education. The exclusion criteria were: (1) currently diagnosed with a disease of the respiratory system, cardiovascular system, or nervous system; (2) anti-social personality disorder or borderline personality disorder; and (3) unwilling to accept the assigned intervention conditions. All participants completed a demographic questionnaire and physical fitness test. Table 1 presents the participant demographics.

**TABLE 1:** Demographics of the participants at baseline.

	TC group	HIIT group	*T*-value	*P*-value
	(*n* = 24)	(*n* = 23)		
Age (years)	31.0 (5.0)	32.7 (4.2)	–1.234	0.224
Height (cm)	174.1 (5.6)	172.4 (6.8)	0.915	0.365
Weight (kg)	77.6 (7.5)	78.0 (11.9)	–0.153	0.879
BMI (kg/m^2^)	25.6 (2.4)	26.2 (3.6)	–0.681	0.131
Years of education (years)	9.1 (2.7)	8.7 (2.2)	0.378	0.209
Years of drug use (years)	6.8 (4.3)	9.0 (4.8)	–1.622	0.112
Age of first drug use (years)	25.1 (5.0)	25.7 (4.6)	–0.435	0.666
Systolic Blood Pressure (mmHg)	119.6 (10.9)	121.6 (16.8)	–0.481	0.633
Diastolic Blood Pressure (mmHg)	67.9 (9.0)	69.9 (9.7)	–0.731	0.469
Vital capacity (ml)	4548.0 (928.5)	4594.0 (940.0)	–0.169	0.867
Hand grip (kg)	52.3 (6.1)	52.0 (9.0)	0.129	0.898
Push up (Repetitions)	32.2 (18.7)	34.0 (9.3)	–0.433	0.693
Sit-and-reach (cm)	13.6 (9.1)	16.5 (8.7)	–1.100	0.277
One-leg stand with eyes closed (s)	47.2 (32.9)	30.0 (20.8)	2.174*	0.038*
Choice reaction time (ms)	485.0 (61.2)	479.6 (58.0)	0.312	0.756

Data presented as mean (SD). TC, Tai chi; HIIT, High-intensity interval training. *p < 0.05.

### Sample size calculation

The participants of our study were screened and the final sample size was 47. To test whether the sample size of ANOVA is reasonable, G*power 3.1 (Franz faul, Universitat Kiel, Germany) was used to conduct the *post hoc* statistical power analysis. Referring to the parameter settings in previous study (Menglu et al., 2021), the ANOVA test was selected, the effect size was set as 0.23, the significance level as 0.05, the sample size as 47, the number of groups as 2, and the number of repeated measurements as 6, the calculated power value was 0.99, exceeding the basic level by 0.80. Therefore, the sample size of this study complied with the requirements.

### Cognitive tasks

#### Go/No-go task

A modified Go/No-go task in which two different stimuli (inverted triangle-No-go stimulus or positive triangle-Go stimulus; side length, 7 cm) were focally presented on a computer screen with a gray background (brightness, 60 CD/m^2^) was employed. The stimulus sequence consisted of 200 stimuli and showed inverted or positive triangles with equal probability. One stimulus appeared randomly in each trial, and the number of consecutive occurrences of each same stimulus was less than four times. The stimuli were presented for 100 ms with variable inter-stimulus intervals (1,000–2,000 ms; average, 1,500 ms) to eliminate the expectation effect of the participants. The participants were asked to press the “F” key with their thumbs as soon as possible when presenting the positive triangle (Go stimulus) but not when presenting an inverted triangle (no go stimulus).

#### Stroop task

A modified computerized Stroop test (Stroop, 1935) was used in this study. The stimuli were four Chinese characters, namely, “red” (红), “yellow” (黄), “green” (绿), and “blue” (蓝), and one neutral symbol, “XX.” Three conditions, namely, congruent, incongruent and neutral, were established. In the congruent condition, the font color was identical to the character meaning; in the incongruent condition, the font color was different from the character meaning; in the neutral condition, the font color was identical to the symbol. The participants were asked to respond to the color of the characters or symbols and ignore the meaning of the words. Participants provided their responses by response key mapping (i.e., red, A key; yellow, S key; green, K key; blue, L key). In the experiment, each participant was allowed 240 trials. Words of each color and symbols of each color were presented 48 times and 12 times respectively in random order. The participants were required to distinguish the color of the stimulus and make the corresponding key reaction on the premise of ensuring the correct response. The stimulating words were displayed on a computer screen for 1,000 ms and disappeared when the response key was pressed. The next stimulus was displayed after 500 ms.

Participants did not speak during the Go/No-go and Stroop tasks to avoid anthropic factors resulting from speaking. Response time (RT) and response accuracy were recorded for all tasks. Cognitive tasks were programmed by E-prime 3.0 (Psychology Software Tools, Inc, Sharpsburg, PA, USA).

Recent studies have suggested that the Go/No-go and Stroop tasks could be used to evaluate inhibitory control (Stroop, 1935; Dalley et al., 2011; Smith et al., 2014). The Go/No-go task focuses on measuring motor response inhibition, while the Stroop task measures a more cognitive form of inhibition called interference inhibition (Wostmann et al., 2013). Most of the available studies employed RT and response accuracy as the primary measures of interest (Smith et al., 2014; Chu et al., 2015, 2017; Tsukamoto et al., 2016). Taking the Go/No-go task test as an example, participants must respond to “Go” stimuli quickly while suppressing responses to “No-go” stimuli accurately; this task requires inhibitory control to overcome the trend of automatic response. In this paradigm, RT refers to the average RT of all correct stimuli, and response accuracy refers to the number of stimuli to which the correct response was given relative to the total number of stimuli. A shorter RT or higher response accuracy or both indicates stronger inhibitory control (Smith et al., 2014).

### Exercise intervention

The participants were randomly assigned by a computer to the TC (*n* = 24) or the HIIT group (*n* = 23) and participated in a single session of exercise. The participants were equipped with a heart rate (HR) monitor (Polar TeamPro, Finland) and then engaged in a 30 min session of TC or HIIT. The TC protocol consisted of a 5 min warm-up, 20 min of modified TC (Zhu et al., 2016) at 55%–75% maximum heart rate (HR_*max*_), and a 5 min cool-down. The HIIT protocol includes a 5 min warm-up, 20 min of interval exercise, and a 5 min cool-down. The participants performed warm-up and cool-down at a self-determined speed on a treadmill (SH-5921, Shangqiu, China) and completed five cycles of 2 min of running at 85–95% HR_*max*_ (estimated as 220 - age) separated by 2 min of self-paced walking. Participants in the HIIT group were allowed to quit the test or ask the experimenter to change the running speed if they felt inadaptable. Ten HR indicators were recorded every 2 min during the 20 min exercise phases of both groups, and the average HR was recorded. Ratings of perceived exertion (RPE, scale of 6–20) (Borg, 1970) were assessed every 5 min.

### Procedure

The participants were informed of the purpose of this study and requested to sign a consent form during the admission process. Laboratory visits for participants were performed on two separate occasions at the same time of day.

During the baseline laboratory visit, the participants were asked to complete a demographics questionnaire, and their physical fitness (Table 1) was evaluated using a model of the Fitness Assessment System (BW-FC-9201L). All tests were repeated two times, and the best score was recorded. The participants were asked to perform the two cognitive tasks in a fixed sequence, starting with the Go/No-go test, under the guidance of experts.

**FIGURE 1:**
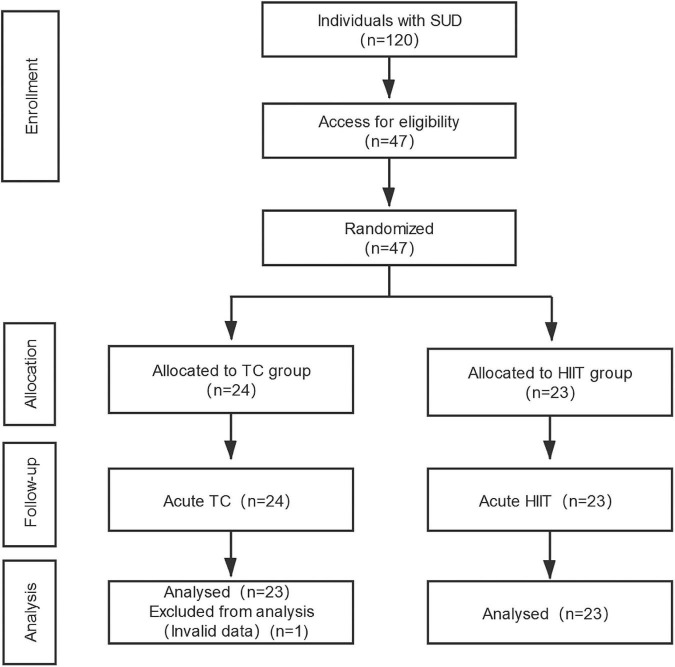
Flow diagram of the intervention process of the two groups.

During the second laboratory visit, the participants were equipped with a HR monitor throughout the intervention session. After completing 30 min of either TC or HIIT, the participants were asked to perform cognitive tasks when their HR returned to less than 10% of their pre-exercise HR. Figure 1 shows the flow diagram of the intervention processes of the two groups.

### Statistical analyses

Statistical analyses were performed using SPSS 22.0 (Chicago, IL, USA). All variables were normally distributed tested with Kolmogorov-Smirnov test, the independent sample *t* test was used for baseline comparison. The results of the Go/No-go test were analyzed by 2 (Time: baseline, post-test) × 2 (Group: TC, HIIT) repeated-measures analysis of variance (ANOVA) to compare differences in Go RT. Response accuracy was further analyzed using 2 (Time: baseline, post-test) × 2 (Group: TC, HIIT) × 2 (Condition: Go, No-go) mixed-design ANOVA. RT and response accuracy in the Stroop test data were analyzed by using 2 (Time: baseline, post-test) × 2 (Group: TC, HIIT) × 3 (Condition: congruent, neutral, incongruent) mixed-design ANOVA. A *post hoc* test with Bonferroni adjustment was used for appropriate pairwise comparison if ANOVA showed a significant interaction. Trials with an RT of <100 ms were excluded from the calculation of participants’ mean correct RT (*n* = 1 test excluded) because <100 ms is considered insufficient for visual processing and stimulus response. All data are presented as the mean ± standard deviation (SD). Statistical significance was set to a *p* < 0.05 according to Fisher statistics (Gigerenzer, 2004). Additionally, the effect size (ES) of differences was analyzed using Neyman–Pearson statistics (Neyman and Pearson, 1992; Nuzzo, 2014).

## Results

### Demographic

Demographics of the participants are presented in Table 1. The participants in the two groups showed no significant differences in terms of age, height, weight, education level, years of drug use, and age of first drug use. While scores for one-leg stand with eyes closed differed between groups, differences in the general physical fitness of the two groups were not significant.

### Cognitive behavioral outcomes

#### Go/No-go test

The descriptive statistical results are shown in Table 2.

**TABLE 2:** Behavioral data for the Go/No-go task.

Variable	TC group (*n* = 23)		HIIT group (*n* = 23)	
	Baseline	Post-test	Baseline	Post-test
Go accuracy (%)	99.3 ± 1.3	99.3 ± 1.0	99.3 ± 0.9	99.4 ± 1.3
No-go accuracy (%)	96.2 ± 2.8	97.8 ± 2.4	96.0 ± 3.8	97.5 ± 2.4
Go RT (ms)	400.9 ± 57.5	377.0 ± 53.0	386.0 ± 40.5	375.4 ± 38.9

Data presented as mean ± SD. TC, Tai chi; HIIT, high-intensity interval training; RT, reaction time.

**FIGURE 2:**
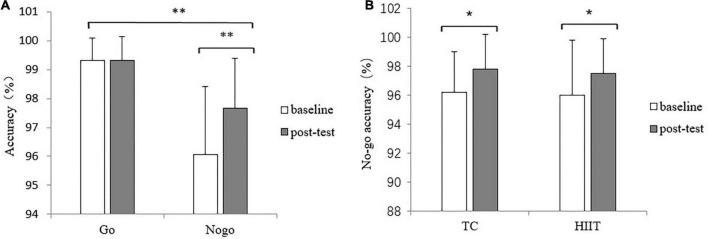
Changes in the accuracy of the Go/no-go test after TC and HIIT: **(A)** overall accuracy of two groups of baseline and post-test, **(B)** No-go accuracy of two groups of baseline and post-test. *p < 0.05, **p < 0.01.

The Go RT evaluated by two-way repeated-measures ANOVA revealed a statistically significant difference in the main effect of time [*F*_(1_,_44)_ = 8.424, *p* = 0.006, η*^2^_*p*_* = 0.161]. The result showed that the Go RT of the post-test (376.2 ± 45.9ms) was lower than that of the baseline (393.5 ± 49.7ms). The interaction between time and group was not significant [F_(1_,_44)_ = 1.229, *p* = 0.274, η*^2^_*p*_* = 0.027]. Additionally, the 2 (Time: baseline, post-test) × 2 (Group: TC, HIIT) × 2 (Condition: Go, No-go) of the accuracy with ANOVA revealed a statistically significant difference in the main effects of time [*F*_(1_,_44)_ = 7.813, *p* = 0.008, η*^2^_*p*_* = 0.151] and condition [*F*_(1_,_44)_ = 53.639, *p* < 0.001, η*^2^_*p*_* = 0.549]. The results showed that the accuracy of the baseline (97.7 ± 1.3%) was lower than that of the post-test (98.5 ± 1.0%) and that the accuracy of the No-go condition (99.3 ± 0.6%) was lower than that of the Go condition (96.9 ± 1.6%). The interaction between time and condition revealed a statistically significant difference [*F*_(1_,_44)_ = 8.866, *p* = 0.005, η*^2^_*p*_* = 0.168]. Simple-effect analysis indicated that the accuracy of the baseline under the No-go condition is lower than that of the post-test (*p* = 0.003), but no significant differences was found between baseline and post-test for the go accuracy (*p* = 0.657); the accuracy at post-test of both TC and HIIT groups under No-go condition was significantly higher than that of the baseline (*p* = 0.028 and *p* = 0.037). The results are shown in Figure 2.

#### Stroop test

The descriptive statistical results are shown in Table 3.

**TABLE 3:** Behavioral data for the Stroop task.

Variable	TC group (*n* = 23)		HIIT group (n = 23)	
	Baseline	Post-test	Baseline	Post-test
congruent accuracy (%)	91.7 ± 4.4	92.5 ± 4.5	92.1 ± 4.8	94.6 ± 3.4
incongruent accuracy (%)	88.6 ± 6.2	91.0 ± 5.4	89.5 ± 6.1	92.8 ± 4.6
neutral accuracy (%)	89.2 ± 6.3	90.7 ± 5.2	89.1 ± 7.1	95.2 ± 3.7
congruent RT (ms)	604.0 ± 52.8	576.4 ± 50.3	591.3 ± 53.0	552.6 ± 40.7
incongruent RT (ms)	634.2 ± 50.7	605.2 ± 48.6	623.0 ± 50.9	575.7 ± 45.2
neutral RT (ms)	623.8 ± 60.0	602.2 ± 46.3	616.4 ± 65.7	567.7 ± 44.9

Data presented as mean ± SD. TC, Tai chi; HIIT, high-intensity interval training; RT, reaction time.

**FIGURE 3:**
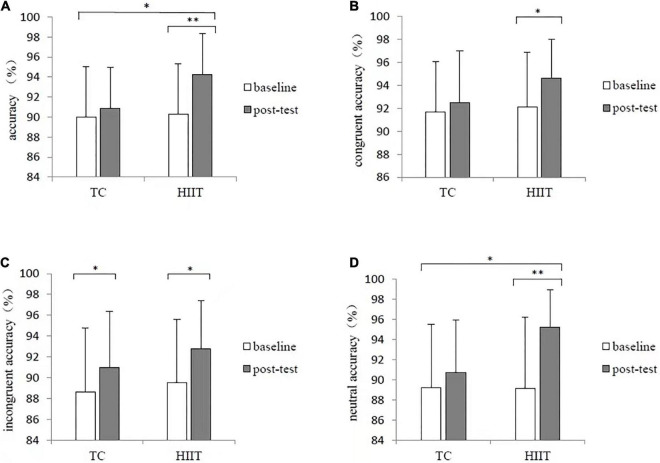
Changes in the four accuracy conditions of the Stroop test after TC and HIIT: **(A)** accuracy in all conditions, **(B)** congruent accuracy, **(C)** incongruent accuracy, and **(D)** neutral accuracy. *p < 0.05, **p < 0.01.

The accuracy evaluated by three-way mixed-design ANOVA revealed a significant difference in the main effects of time [*F*_(1_,_44)_ = 21.643, *p* < 0.001, η*^2^_*p*_* = 0.330] and condition [*F*_(2_,_88)_ = 8.598, *p* < 0.001, η*^2^_*p*_* = 0.163]. The results showed that the accuracy at the baseline (90.1 ± 3.6%) was lower than that of the post-test (92.8 ± 2.6%); the accuracy of the incongruent condition (90.5 ± 3.6%) was lower than those of the congruent condition (92.8 ± 2.4%) and neutral condition (91.1 ± 3.4%). The interaction between time and group revealed a significant difference [*F*_(1_,_44)_ = 4.162, *p* = 0.047, η*^2^_*p*_* = 0.086]. *Post hoc* test indicated that the accuracy of the post-test of HIIT group was significantly higher than that of the TC group (*p* < 0.001). Sample effect test showed that the congruent accuracy at post-test of HIIT group was significantly higher than that of the baseline (*p* = 0.025); the incongruent accuracy at post-test of both TC and HIIT groups were significantly higher than that of the baseline (*p* = 0.030 and *p* = 0.003); the neutral accuracy at post-test of HIIT was significantly higher than that of the baseline (*p* < 0.001) and TC group (*p* = 0.002). The results are shown in Figure 3.

The RT determined by three-way mixed-design ANOVA revealed a significant difference in the main effects of time [*F*_(1_,_44)_ = 39.738, *p* < 0.001, η*^2^_*p*_* = 0.475] and condition *F*_(2_,_88)_ = 73.684, *p* < 0.001, η*^2^_*p*_* = 0.626]. The interaction between time and condition was not significant [*F*_(2_,_88)_ = 0.887, *p* = 0.410, η*^2^_*p*_* = 0.020]. The results showed the RT of the post-test (580.0 ± 31.9 ms)was lower than that of the baseline (615.5 ± 39.1 ms), and the RT of the incongruent condition (609.5 ± 33.6 ms) was higher than those of the congruent (581.1 ± 32.1 ms) and neutral conditions (602.5 ± 35.4 ms).

### Exercise outcomes

**FIGURE 4:**
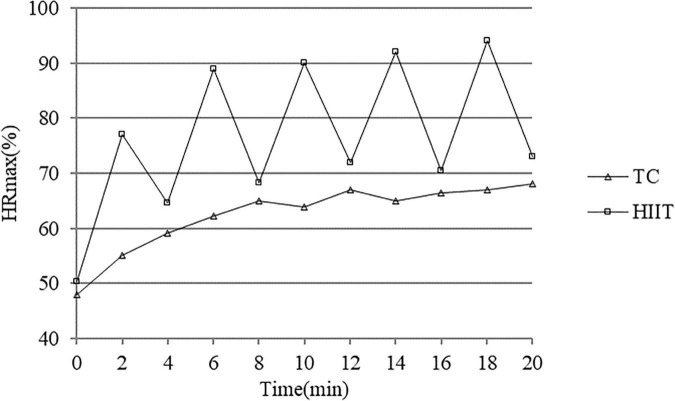
HR_max_ observed during each exercise condition.

The overall average HRs of individuals with SUD during the TC and HIIT sessions were 121 ± 8 bpm and 148 ± 21bpm, respectively. The average HR range of individuals with SUD in the TC group was 55–75% HR_max_, which corresponds to the range of HRs following moderate-intensity exercise [60–70% HR_max_ (Chu et al., 2017; Kao et al., 2017)]. The average HR in the HIIT group during the running period was 165 ± 12 bpm, which is 75–95% HR_max_. The average HR during the interval period was 130.20 ± 6.27 bpm, which is 65–75% HR_max_. These values have been proved to elicit 90–95% HR_max_, which is consistent with the HIIT protocol (Heisz et al., 2016). The RPE scores observed during the TC and HIIT sessions were 13 ± 1 and 16 ± 2, respectively. Independent sample *t*-test revealed significant differences in exercise HR (*t* = -3.878, *p* < 0.001) and RPE score (*t* = -8.756, *p* < 0.001) between the two groups, thus suggesting that the exercise sessions achieved the appropriate intensities. HR_max_ values observed under each exercise type are shown in Figure 4.

## Discussion

In this study, the acute effects of TC and HIIT on inhibitory control were studied using behavioral methods during the Go/No-go and Stroop tasks. The findings indicated that TC and HIIT affect performance in the two cognitive tasks differently. In the Go/No-go test, acute TC and HIIT resulted in shorter RTs compared with the baseline and selectively increased response accuracy for incongruent trials. In the Stroop test, acute TC and HIIT resulted in shorter RTs under incongruent, neutral, and congruent conditions compared with the baseline. Moreover, the HIIT group showed superior improvements in the accuracy of cognitive inhibition under incongruent, neutral, and congruent conditions, while the TC group indicated improved accuracy only for incongruent trials. Thus, we summarize our findings under two main themes: (1) Improvements in inhibitory control and (2) additional cognitive benefits from HIIT.

### Improvements in inhibitory control

The behavioral outcomes showed that the accuracy of both groups in the Go/No-go test improved and that RTs in the Go/No-go and Stroop tests were shorter in the number of trials that needed to be inhibited (under incongruent conditions). Inhibitory control is generally considered to be related to changes in RT and response accuracy under the incongruent conditions of the Go/No-go and Stroop tests (Cohen et al., 1990; Yanagisawa et al., 2010; Wang et al., 2015). The results demonstrated that both types of exercise lead to increased cognitive performance in SUD individuals, regardless of Go/No-go or Stroop congruency, thereby suggesting that TC and HIIT can improve the behavioral inhibitory control of SUD individuals. This work is the first to provide evidence that TC and HIIT have similar benefits on the cognition of SUD individuals after acute exercise. Although the study lacked a blank control group, we examined several studies to eliminate the improvement of task operation caused by familiar cognitive tasks. The studies reported that the control group (non-exercise group) did not improve cognitive task processing ability (Xiang, 2018; He et al., 2021), which might help indicate that the results of the cognitive improvement can be considered as the specific effects of TC or HIIT. TC is a Chinese traditional body–mind exercise. Although previous studies reported that TC is an exercise that can effectively improve the physical and mental health of SUD individuals (Wang et al., 2014; Zhu et al., 2016, 2018), evidence of the ability of TC to improve the cognitive functions of individuals with SUD is unclear. The benefits of TC in elderly or cognitively impaired groups have been confirmed (Lam et al., 2012; Mortimer et al., 2012; Yang et al., 2015). Previous research showed that long-term regular practice of TC could alleviate declines in the cognitive ability of the elderly (Lam et al., 2012). However, one study found that TC does not improve the cognitive ability of the elderly (Gerritsen et al., 2020). Further research is needed to understand the differences in the boundary conditions of these contradictory effects. Because the intensity of TC was not monitored in the article (Gerritsen et al., 2020) and may have been too low or too high, the results of cognitive improvement may not have reached statistical significance. In general, TC is considered a low-to-moderate-intensity exercise (Pan et al., 2013; Hawkes et al., 2014; Zhu et al., 2016) that results in HR_max_ of approximately 50–70% (Lan et al., 1998; Cui et al., 2019). However, no comparison of the different exercise intensities of TC and the corresponding cognitive benefits is yet available. According to ACSM’s Guidelines for Exercise Testing and Prescription (Thompson et al., 2013), the HR range of low-intensity exercise is 57–64% HR_max_, that of moderate-intensity exercise is 64–76% HR_max_, and that of high-intensity exercise is 76–96% HR_max_. Thus, the intensity of the TC in our study can be considered moderate (HR range = 55–75% HR_max_, average HR = 64% HR_max_). Earlier studies revealed that low-intensity exercise is not effective in improving cognitive function (McMorris and Hale, 2012; Swenson et al., 2020). Prior studies have demonstrated that a single 20–30 min bout of moderate intensity exercise facilitated cognitive performance (Kamijo et al., 2007, 2009). Overall, cognitive changes due to TC may be related to exercise intensity. Our study supports previous findings that moderate-intensity TC provides cognitive improvements in individuals with SUD and suggests that this exercise may be effective for improving cognitive conditions in SUD individuals.

In contrast to TC, HIIT is a vigorous exercise (Gayda et al., 2016). Some studies revealed that HIIT could improve the inhibitory control of the cognitive impairment group (Lautenschlager et al., 2008; Baker et al., 2010; Fiorelli et al., 2019). Other studies have indicated that the inhibitory control benefits of HIIT and MICT are similar (Kao et al., 2018), consistent with our findings in this study. To date, few articles exploring cognitive inhibitory control due to TC and other sports are available, and the current research cannot yet explain the advantages and disadvantages of moderate-intensity TC and other-intensity sports in detail. This article may help explain the difference between moderate-intensity TC and high-intensity interval exercises.

### Additional cognitive benefits from high-intensity interval training

While no difference in RT was found between the two groups, the HIIT group showed superior improvements in cognitive accuracy under congruent and neutral conditions compared with the TC group in the Stroop task. By comparison, the accuracy of the TC group under these conditions simply indicated an upward trend. Given this finding, HIIT may be better able to improve cognitive accuracy than TC. However, the results do not show the difference between the two groups under incongruent conditions, and the accuracy rate of groups under incongruent conditions increased significantly in the *post hoc* test. The increase in response accuracy under HIIT in congruent and neutral conditions may not indicate that HIIT results in better inhibitory control, given that inhibitory control is generally considered to be related to changes in RT and response accuracy under the incongruent condition of the Stroop test (Cohen et al., 1990). More accurately, it may be that general cognition is improved. This work is the first to compare the cognitive effects of HIIT with TC, which are two completely different forms of exercise. Our study found that HIIT seems to be slightly better than TC in improving general cognitive accuracy. Although no direct evidence supports the results above, considering that TC is a moderate-intensity exercise, indirect evidence suggests similar behavioral outcomes (Tsukamoto et al., 2016; Kao et al., 2018). Exercise can improve the inhibitory control of patients with SUD (Wang et al., 2016; Swenson et al., 2020). Principally, the effects of different intensities of exercise seem to be different. According to the inverted U-shape relationship between exercise intensity and cognition (Davey, 1973), moderate-intensity exercise appears to provide greater cognition benefits than low- or high-intensity exercise. However, this general relationship does not consider the individual’s initial conditions. Especially in individuals with SUD, the intensity of intervention could be highly dependent on the stage of recovery. In the laboratory setting, recent studies have found that HIIT tends to be more beneficial to inhibitory control than MICT (Tsukamoto et al., 2016; Kao et al., 2017). Our results are consistent with a prior study (Alves et al., 2014) that showed that inverted-U theory is unsuitable for explaining the effects of HIIT. In brain-function studies, HIIT evoked cortical activation related to a Stroop interference on the left-dorsal-lateral prefrontal cortex, which corresponds with improved inhibitory control (Kujach et al., 2018). Furthermore, faster P3 latencies concurrent with smaller P3 amplitudes were found in HIIT compared with MICT, thereby indicating that HIIT may result in more efficient neuroelectric activation, as manifested by faster stimulus identification (Kao et al., 2017). Long-term HIIT training can also improve exercise ability, metabolism, and cardiovascular health more effectively compared with MICT (Tabata et al., 1996; Gayda et al., 2016). In summary, although HIIT showed inhibitory control benefits similar to those obtained from TC in this study, the former provides wider cognitive benefits, which suggests it is a better rehabilitation exercise for SUD. The result from this aspect is consistent with our hypothesis.

Moreover, the similar improvements and differences were also found in individuals with SUD and non-dependent populations in previous studies (Tsukamoto et al., 2016; Cheung et al., 2018; Kao et al., 2018; Wang et al., 2018), indicating that the cognitive improvement of TC and HIIT was not specific to individuals with SUD. Since the cognition of individuals with SUD is worse than that of healthy people (Jones et al., 2016; Fitzpatrick et al., 2020), we believe that TC and HIIT are more meaningful and urgent to improve the cognition of individuals with SUD. Future studies should focus on the specificity of TC or HIIT for the cognitive improvement of individuals with SUD.

Despite the benefits of exercise on cognition, other factors must also be considered for the practical applications of these exercises. Because the safety of HIIT is disputed (Rognmo et al., 2012), TC, the clinical safety of which is well established, may be a better rehabilitation exercise for special groups, such as SUD patients. Combining the results of this study, other factors affecting actual applications, and the different physical rehabilitation advantages brought about by the two exercises (Tabata et al., 1996; Gayda et al., 2016; Zhu et al., 2018), we consider that TC is more suitable for the exercise rehabilitation of patients with SUD in the primary stage and that HIIT may be more suitable for the later stage of exercise rehabilitation. Both activities are feasible exercises that can be combined to contribute to the complete rehabilitation of patients with SUD.

The present study includes a number of limitations that may affect the generalizability of the results. Because we mainly relied on original Fisher statistics extended by ES according to Neyman–Pearson, generalizability cannot be established. The study groups only included male participants, which mean the observed benefits cannot be generalized to females or other cohorts. We set the cognitive tasks test into a set order before and after exercise intervention, which may influence the performance of the participants. The cognitive tasks used in the study involved neutral task paradigms rather than substance-related task paradigms. Because drug abusers show defects in inhibition, the response of drug abusers to drug-related cues may show more obvious activation level than neutral cues (Zamani et al., 2014). Another drawback of this study is the lack of a blank control group or low-intensity exercise group. Moreover, whether the effects in the present study are dependent on the type of movement or the intensity of the exercise requires further research. Future research may be combined with EEG, fNIRS or other neuroimaging techniques to explain the mechanism by which TC or HIIT intervention promotes cognitive function in individuals with SUD.

## Conclusion

The present investigation showed similar short-term facilitative effects on inhibitory control following TC and HIIT in patients with SUD. TC and HIIT can similarly promote the inhibitory control of individuals with SUD. Compared with the TC group, the HIIT group showed greater improvements in response accuracy. These findings demonstrate the potential applications of TC and HIIT in improving cognition in SUD. Both TC and HIIT are worth of being recommended as cognitive rehabilitation exercise for patients with SUD and the results encourage to continue the research according to the original interpretation of Fisher-statistics.

## Author’s note

The manuscript has been submitted as a preprint (Pang et al., 2021).

## Data availability statement

The data presented in this study will be available on request from the corresponding author and must be subject to privacy restrictions.

## Ethics statement

The studies involving human participants were reviewed and approved by Shanghai Narcotics Control Commission Ethical Committees of and the Shanghai University of Sport. The patients/participants provided their written informed consent to participate in this study.

## Author contributions

YY participated in the study design, data analysis, and manuscript revision. XP participated in the study design, data analysis, manuscript draft, and manuscript revision. SY participated in the study design and manuscript revision. KX participated in the coordination of intervention conducted in Shanghai Drug Compulsory Rehabilitation Center and established the appropriate experimental environment. TW and JW participated in the exercise intervention and data collection. WS participated in manuscript revision. DZ participated in the study design, manuscript revision, and coordination of intervention conducted in Shanghai Drug Compulsory Rehabilitation Center. All authors read and approved the final manuscript.
